# Supplementary figures and images for: Multiple mechanisms regulate H3 acetylation of enhancers in response to thyroid hormone
Source: PLoS Genet. 2020 May 26;16(5):e1008770. doi: 10.1371/journal.pgen.1008770 (PMC7274477; doi:10.1371/journal.pgen.1008770)

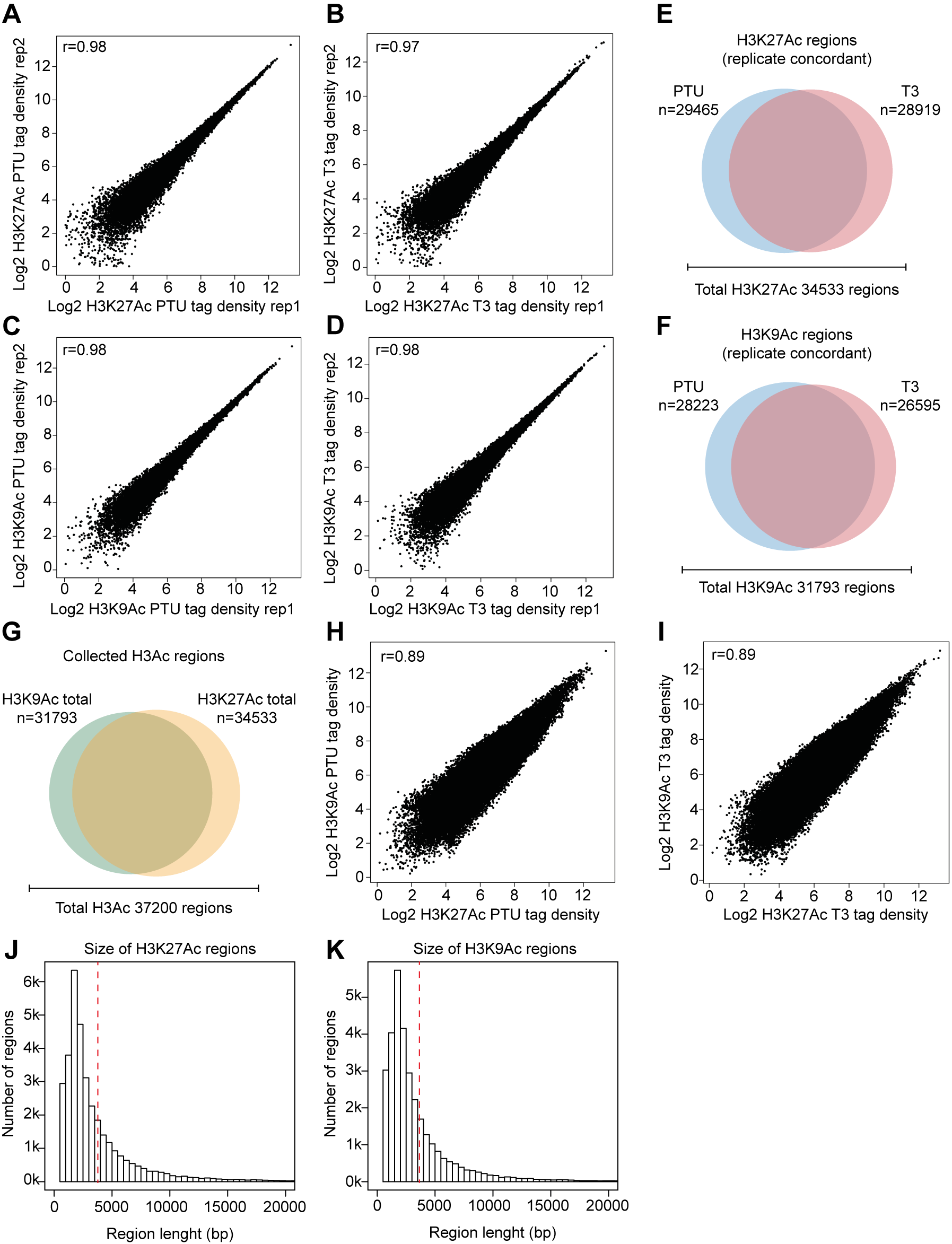

Supplement: S1 Fig — (A) Correlation between two replicate H3K27Ac ChIP-seq experiments in mice treated with PTU. (B) Correlation between two replicate H3K27Ac ChIP-seq experiments in mice treated with PTU+T3. (C) Correlation between two replicate H3K9Ac ChIP-seq experiments in mice treated with PTU. (D) Correlation between two replicate H3K9Ac ChIP-seq experiments in mice treated with PTU+T3. (E) Replicate concordant H3K27Ac regions in mice treated with PTU and PTU+T3. (F) Replicate concordant H3K9Ac regions in mice treated with PTU and PTU+T3. (G) Combined number of identified H3K27Ac and H3K9Ac regions. (H) Correlation between H3K27Ac and H3K9Ac in mice treated with PTU. (I) Correlation between H3K27Ac and H3K9Ac in mice treated with PTU+T3. (J) Size of identified H3K27Ac regions. (K) Size of identified H3K9Ac regions. (TIF) [file pgen.1008770.s001.tif]

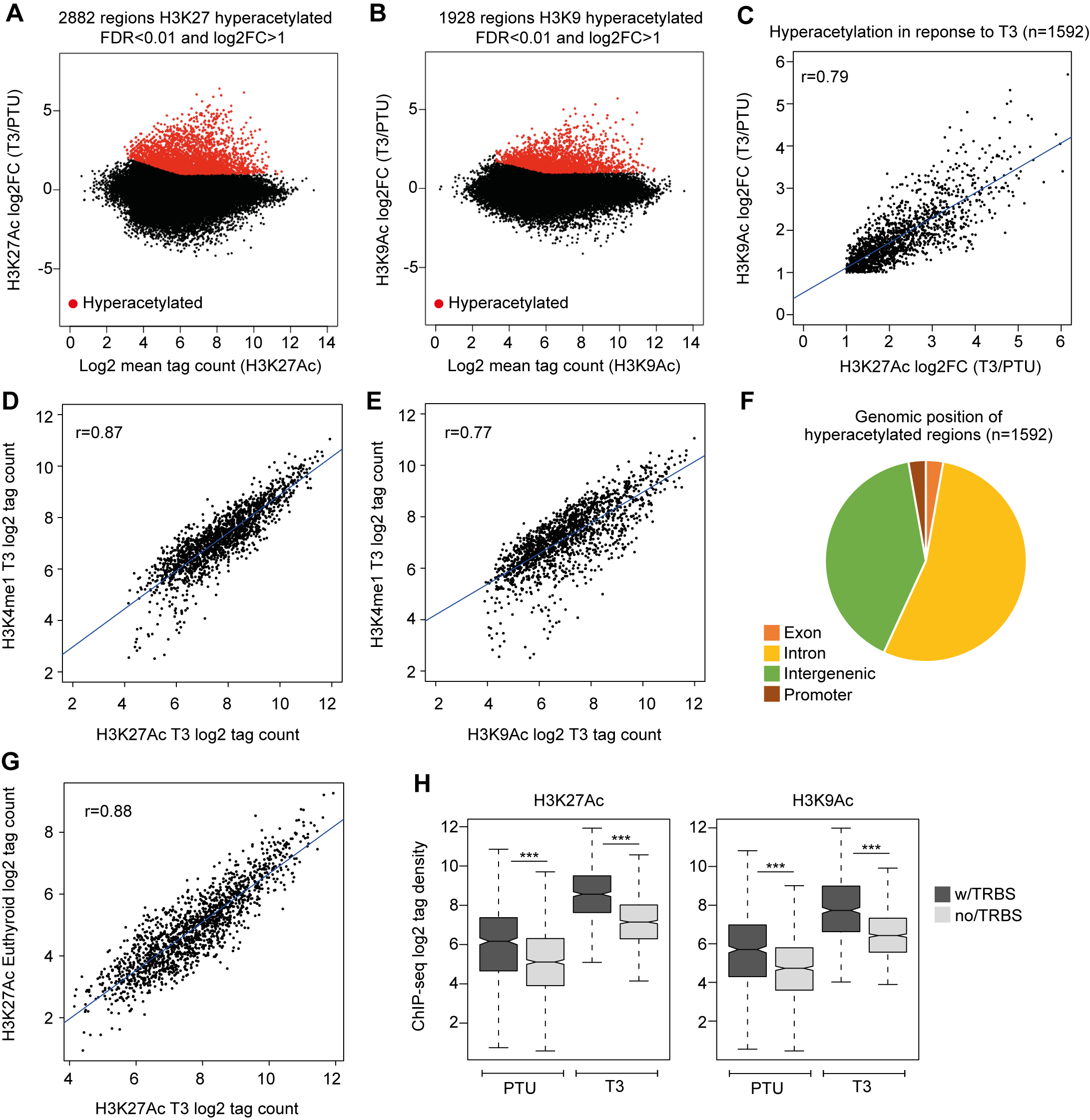

Supplement: S2 Fig — (A) Identification of 2882 H3K27 hyperacetylated regions in hyperthyroid condition (FDR<0.01 and log2FC>1). (B) Identification of 1928 H3K9 hyperacetylated regions in the hyperthyroid condition (FDR<0.01 and log2FC>1). (C) Correlation between H3K27- and H3K9 hyperacetylation at 1592 H3 hyperacetylated regions. (D) Correlation between H3K4me1 and H3K27Ac at hyperacetylated regions (n = 1592). (E) Correlation between H3K4me1 and H3K9Ac at hyperacetylated regions (n = 1592). (F) Distribution of hyperacetylated regions within exons, introns, promoters and intergenic regions. (G) Correlation between H3K27Ac in hyperthyroid and euthyroid condition (n = 1592). Correlation coefficient (Pearson) indicated in plots panels C, D, E and G. (H) Quantification of H3K27Ac and H3K9Ac at regions hyperacetylated with (w/TRBS) and without TRBS (no/TRBS) in response to T3. Statistical difference was determined by a Wilcoxon Signed Rank Test, ***p<0.001. (TIF) [file pgen.1008770.s002.tif]

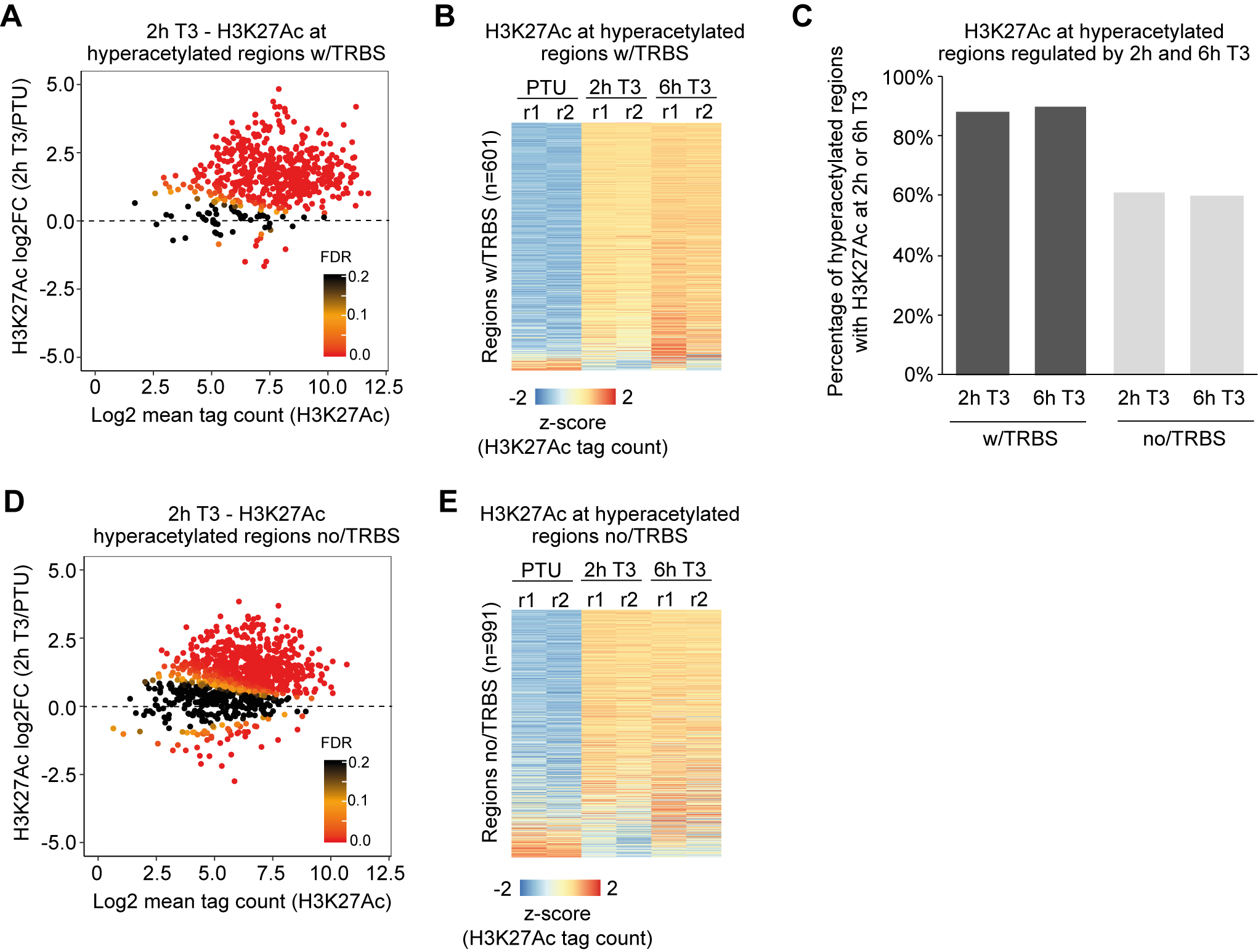

Supplement: S3 Fig — (A) H3K27Ac after 2h of T3 treatment was quantified at hyperacetylated regions with a TRBS (w/TRBS) and analysed by DESeq2. FDR<0.05 are coloured red. (B) Quantification of H3K27Ac in response to 2h and 6h treatment with T3 at hyperacetylated regions with a TRBS. ChIP-seq tag counts are normalized by a z-score. (C) Percentage of hyperacetylated regions with significant increased H3K27Ac (FDR<0.05, Log2FC>0) after 2h and 6h treatment with T3. (D) H3K27Ac after 2h of T3 treatment was quantified at hyperacetylated regions without a TRBS (no/TRBS) and analysed by DESeq2. FDR<0.05 are coloured red. (E) Quantification of H3K27Ac in response to 2h and 6h treatment with T3 at hyperacetylated regions without a TRBS. ChIP-seq tag counts are normalized by a z-score. (TIF) [file pgen.1008770.s003.tif]

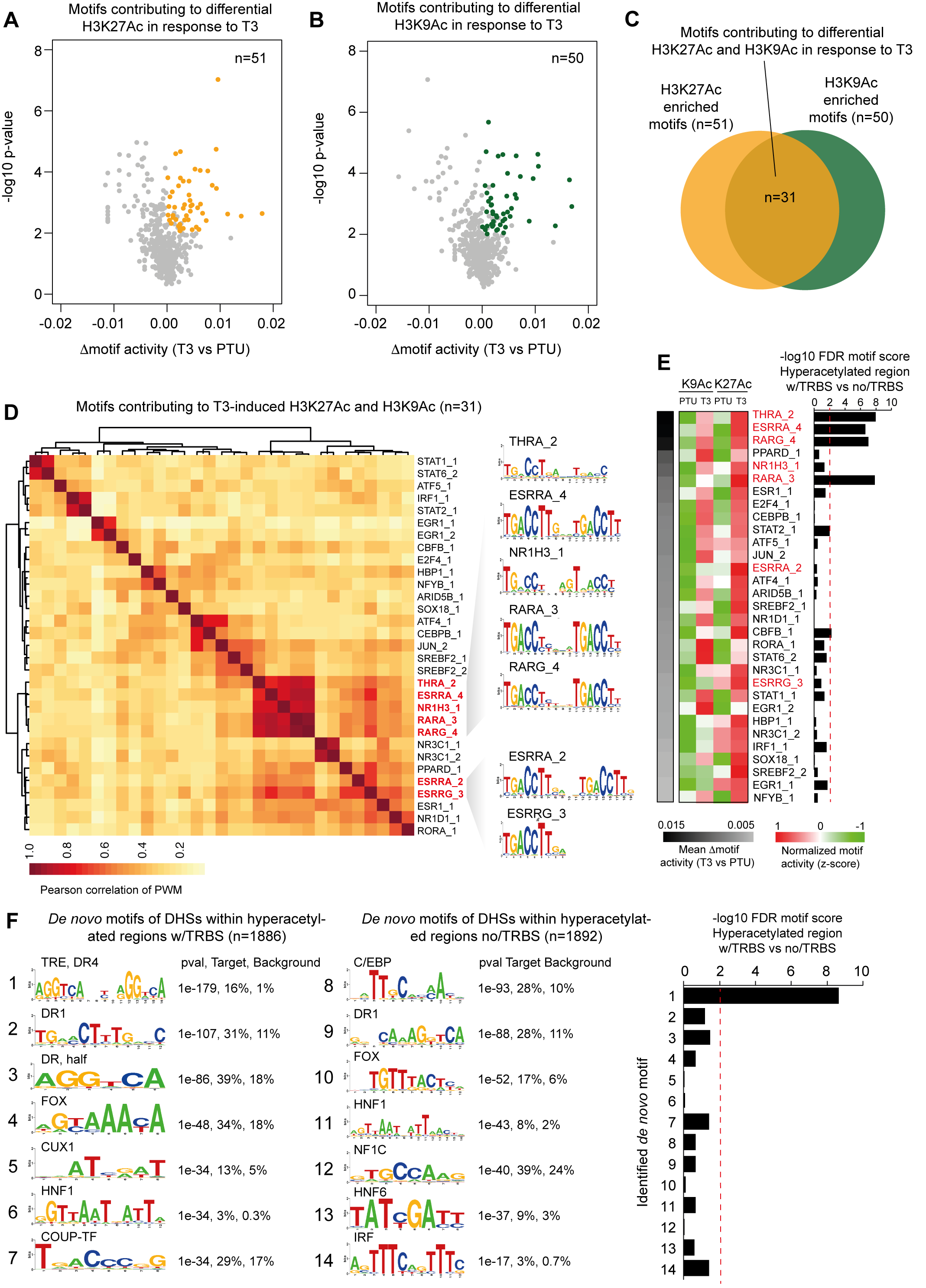

Supplement: S4 Fig — (A) Motifs contributing to T3-regulated H3K27Ac. Motifs contributing to T3-induced H3K27Ac with p<0.01 are coloured yellow. (B) Motifs contributing to T3-regulated H3K9Ac. Motifs contributing to T3-induced H3K9Ac with p<0.01 are coloured green. (C) Motifs contributing to both H3K27 and H3K9 hyperacetylation by T3. (D) Hierarchical clustering of pearson correlation of the positions weight matrix (PWM) of motifs contributing to T3-induced H3K27 and H3K9 acetylation. Motifs resembling DR4 or DR4 half sites are shown on the right. (E, left) Motifs contributing to T3-induced H3K9Ac and H3K27Ac evaluated by IMAGE analysis. Motifs enriched at p<0.01 are ranked according to the mean differential motif activity (z-score) in response to T3 (Dmotif activity). Normalized motif activities for H3K9Ac and H3K27Ac in hypo- and hyperthyroid condition are visualized as a heatmap. Motifs resembling DR4 or DR4 half site are marked red. (E, right) Statistical test of differential motif scores of hyperacetylated enhancers with and without TRBS. The test was performed using Wilcoxon Signed Rank Test corrected for multiple testing using Benjamini & Hochberg method. (F) De novo DNA motif analysis of DHSs associated with hyperacetylated regions with and without TRBS. Left part of the panel shows statistical test of differential motif scores. The statistical test was performed using Wilcoxon Signed Rank Test corrected for multiple testing using Benjamini & Hochberg method. (TIF) [file pgen.1008770.s004.tif]

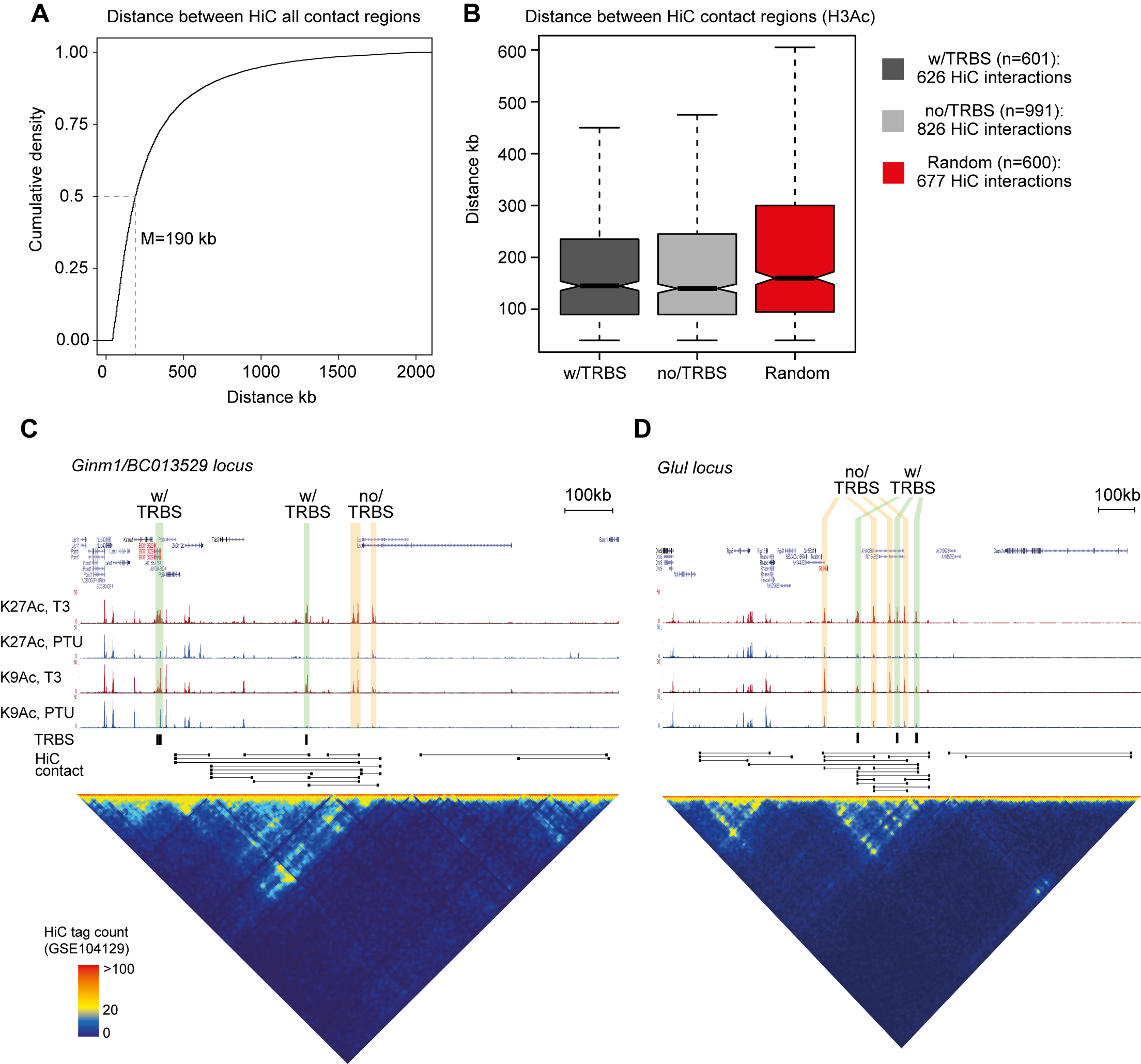

Supplement: S5 Fig — (A) Distance between all interacting regions identified from HiC. (B) Distance between hyperacetylated regions associated with and without TRBSs. (C and D) Examples of interacting regions near T3-regulated genes. Hyperacetylated regions (T3-regulated enhancers) are indicated by green (w/TRBS) and orange (no/TRBS). The T3-regulated Ginm1 and Glul genes are indicated in red. (TIF) [file pgen.1008770.s005.tif]

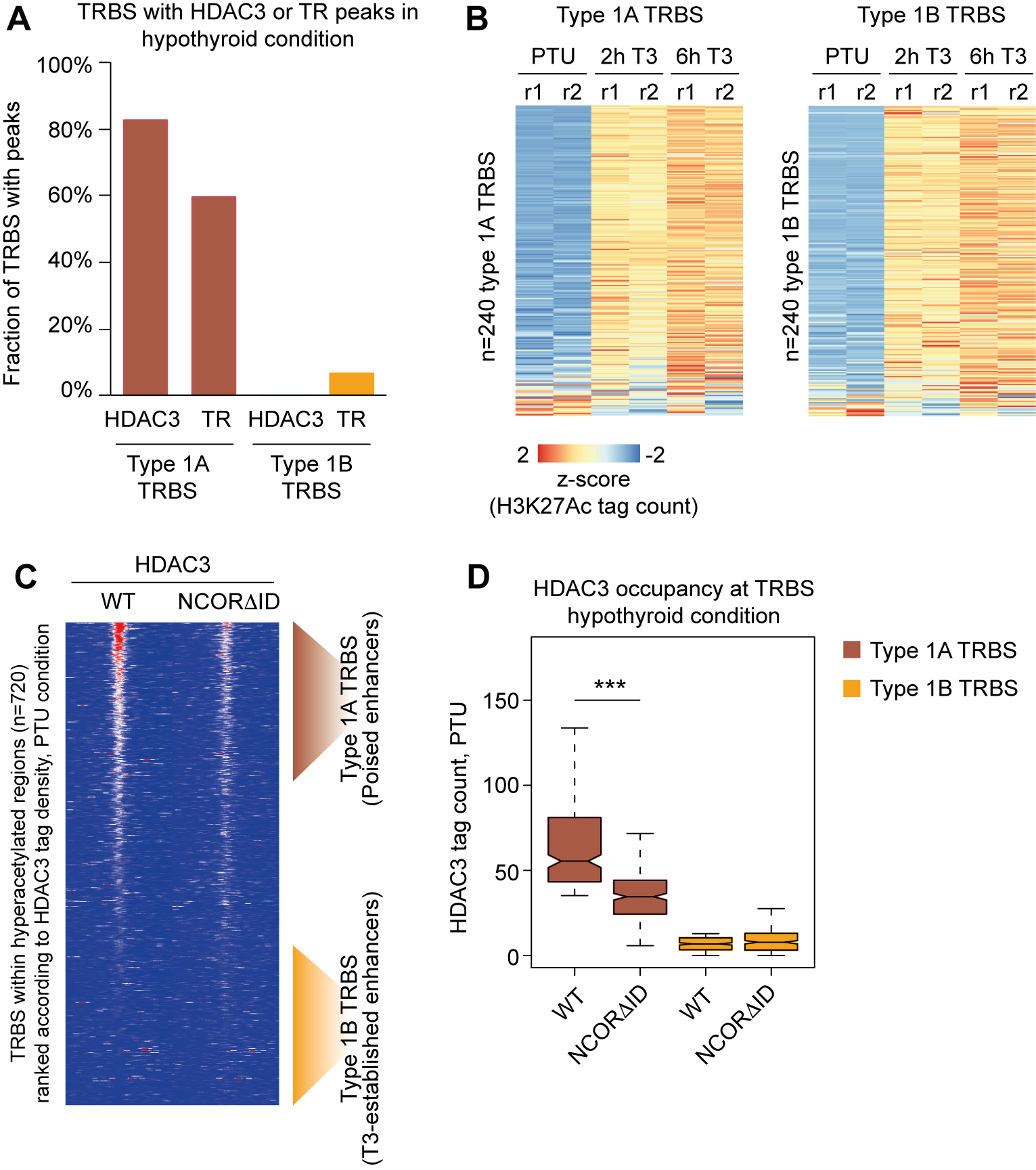

Supplement: S6 Fig — (A) Fraction of type 1A and type 1B TRBS with HDAC3 or TR peaks in hypothyroid condition. (B) H3K27Ac at type 1A and type 1B TRBS in response to 2h and 6h T3 treatment. (C) Heatmap illustrating HDAC3 occupancy at TRBS in the NCORΔID mutant compared to WT. TRBS are ranked according to HDAC3 occupancy in hypothyroid WT mice. HDAC3 ChIP-seq performed on livers from hypothyroid animals. (D) Quantification of HDAC3 occupancy at TRBSs associated with type 1A and type 1B TRBSs. Statistical difference was determined by a Wilcoxon Signed Rank Test, ***p<0.001. (TIF) [file pgen.1008770.s006.tif]
