# Supplementary material for: Multiple mechanisms regulate H3 acetylation of enhancers in response to thyroid hormone
Source: PLoS Genet. 2020 May 26;16(5):e1008770. doi: 10.1371/journal.pgen.1008770 (PMC7274477; doi:10.1371/journal.pgen.1008770)
Supplement: S1 Table — Data represent average with indicated standard deviations. (PDF) [file pgen.1008770.s007.pdf]

Table S1. Age, body weight and liver weight of animals. Data represent average with indicated standard deviations.

Genotypes of mice	n	Age (months)	Body weight(g)	Liver weight (mg)
WT PTU	14	4.97±1.76	23.1±0.6	1325±130
WT PTU+T3	13	4.71±1.31	23.0±1.0	1211±93
WT PTU+T3 (2h)	3	3.03±0.27	24.7±3.1	1289±52
WT PTU+T3 (6h)	3	3.03±0.27	22.7±0.6	1084±83
Thrb$^{PV/PV}$ PTU	3	2.23±1.14	21.5±0.2	1960±423
Thrb$^{PV/PV}$ PTU+T3	3	2.27±1.01	20.9±0.8	1769±134
NCOR1$^{\Delta ID/\Delta ID}$ PTU	7	3.11±1.29	24.2±3.1	1613±243
