# Supplementary material for: Multiple mechanisms regulate H3 acetylation of enhancers in response to thyroid hormone
Source: PLoS Genet. 2020 May 26;16(5):e1008770. doi: 10.1371/journal.pgen.1008770 (PMC7274477; doi:10.1371/journal.pgen.1008770)
Supplement: S2 Table — Primers used in HDAC3, NCOR1, CBP, SRC1, MED1 and p300 ChIP-qPCR experiments presented in Fig 3E. (PDF) [file pgen.1008770.s008.pdf]

Table S2. ChIP-qPCR primers. Primers used in HDAC3, NCOR, CBP, SRC1, MED1 and p300 ChIP-qPCR experiments presented in Figure 3E.

ID	Target region	Forward	Reverse
Ctrl	chr15:74,127,392-74,127,442	TGGTAGCCTCAGGAGCTTGC	ATCCAAGATGGGACCAAGCTG
1	chr2:25,328,074-25,328,155	CATAGCTTCTCCCCAAATCCCCTCC	GGCACCTCTGTCCTGCTCAGATAAT
2	chr2:69,224,332-69,224,422	CAGTTCATGGAAGAGTGGTT	CATAGAGAAAGGGACAGCCA
3	chr4:131,790,802-131,790,924	GTCCAGAGTCCATCATCTTC	GGCTTCTCTAATCTTCGTCA
4	chr17:28,416,156-28,416,265	GCTGCCAGAGCTTTTATTTTC	CAAAAACCAGAAAGAGCAGG
5	chr9:42,143,019-42,143,104	GTGTCTTCCTCTGTCTCTTG	CAGGTGTGTTATGGAGTTTC
6	chr4:154,691,237-154,691,323	TGTGTAAACACCACCTTTGA	ACTTGGAATACTGGACCCAC
7	chr14:71,096,444-71,096,529	CAGTTGTGGGTGAGTTTGAG	TTCCAGGCATCAGGAAAGAA
8	chr15:7,201,868-7,201,993	GATGTGTTCCTTCTATTTGTCC	CCTTAGCTGACTTAGTGGTG
