# Supplementary material for: Multiple mechanisms regulate H3 acetylation of enhancers in response to thyroid hormone
Source: PLoS Genet. 2020 May 26;16(5):e1008770. doi: 10.1371/journal.pgen.1008770 (PMC7274477; doi:10.1371/journal.pgen.1008770)
Supplement: S3 Table — Sequenced tags were aligned to mm9 using STAR. Uniquely aligned reads were used for downstream analysis. (PDF) [file pgen.1008770.s009.pdf]

Table S3. Summary of next generation sequencing data.

Sequenced tags were aligned to mm9 using STAR. Uniquely aligned reads were used for downstream analysis.

Treatment & Genotype	Type of experiment	Total reads	Uniquely aligned reads	Data used in fig.
PTU WT, rep1	H3K27Ac ChIP-seq	31334225	28953122	Fig1+2+3+4
PTU+T3 WT, rep1	H3K27Ac ChIP-seq	33703644	30808912	Fig1+2+3+4
PTU NCORΔID, rep1	H3K27Ac ChIP-seq	51701843	47244843	Fig4
PTU WT, rep2	H3K27Ac ChIP-seq	41879515	38525577	Fig1+2+3+4
PTU+T3 WT, rep2	H3K27Ac ChIP-seq	27376407	24827637	Fig1+2+3+4
PTU NCORΔID, rep2	H3K27Ac ChIP-seq	20762559	18838874	Fig4
PTU+T3 2h WT, rep1	H3K27Ac ChIP-seq	31701444	22839387	Fig2
PTU+T3 2h WT, rep2	H3K27Ac ChIP-seq	29788788	22171235	Fig2
PTU+T3 6h WT, rep1	H3K27Ac ChIP-seq	25051412	19591035	Fig2
PTU+T3 6h WT, rep1	H3K27Ac ChIP-seq	21118579	15060480	Fig2
PTU WT, rep1	H3K9Ac ChIP-seq	25698553	23882444	Fig1+2+3+4
PTU+T3 WT, rep1	H3K9Ac ChIP-seq	37768046	34693895	Fig1+2+3+4
PTU NCORΔID, rep1	H3K9Ac ChIP-seq	45025767	41649814	Fig4
PTU WT, rep2	H3K9Ac ChIP-seq	51913259	48224650	Fig1+2+3+4
PTU+T3 WT, rep2	H3K9Ac ChIP-seq	32360571	29655439	Fig1+2+3+4
PTU NCORΔID, rep2	H3K9Ac ChIP-seq	51817291	47750868	Fig4
PTU WT, rep1	H3K27Ac ChIP-seq	11428298	10155231	Fig1
PTU WT, rep2	H3K27Ac ChIP-seq	10219796	8811615	Fig1
PTU+T3 WT, rep1	H3K27Ac ChIP-seq	11875167	10503254	Fig1
PTU+T3 WT, rep2	H3K27Ac ChIP-seq	11239232	10146979	Fig1
PTU PV, rep1	H3K27Ac ChIP-seq	10406940	9447426	Fig1
PTU PV, rep2	H3K27Ac ChIP-seq	10921287	9871289	Fig1
PTU+T3 PV, rep1	H3K27Ac ChIP-seq	10798210	9755167	Fig1
PTU+T3 PV, rep2	H3K27Ac ChIP-seq	11855385	10717502	Fig1
PTU WT, rep1	HDAC3 ChIP-seq	11620203	10318174	Fig3+4
PTU+T3 WT, rep1	HDAC3 ChIP-seq	10824037	9883066	Fig3+4
PTU NCORΔID, rep1	HDAC3 ChIP-seq	11038199	9930846	Fig4
PTU WT, rep2	HDAC3 ChIP-seq	10794980	9589167	Fig3+4
PTU+T3 WT, rep2	HDAC3 ChIP-seq	15277235	14015664	Fig3+4
PTU NCORΔID, rep2	HDAC3 ChIP-seq	10260217	9281780	Fig4
PTU WT	MED1 ChIP-seq	30647539	24413212	Fig3
PTU+T3 WT	MED1 ChIP-seq	29857374	23797995	Fig3
PTU WT, rep1	NCOR1 ChIP-seq	14475422	12002911	Fig3
PTU+T3 WT, rep1	NCOR1 ChIP-seq	13541040	9524313	Fig3
PTU WT, rep2	NCOR1 ChIP-seq	23189200	15824844	Fig3
PTU+T3 WT, rep2	NCOR1 ChIP-seq	24810734	16947676	Fig3
PTU WT, rep1	CBP ChIP-seq	14057091	11774126	Fig3
PTU+T3 WT, rep1	CBP ChIP-seq	10543791	8813856	Fig3
PTU WT, rep2	CBP ChIP-seq	19600376	12094802	Fig3
PTU+T3 WT, rep2	CBP ChIP-seq	22313358	12012890	Fig3
PTU WT, rep1	H3K4me1 ChIP-seq	13200799	11579774	Fig3
PTU+T3 WT, rep1	H3K4me1 ChIP-seq	13419013	11766879	Fig3
PTU WT, rep2	H3K4me1 ChIP-seq	28222064	25660376	Fig3
PTU+T3 WT, rep2	H3K4me1 ChIP-seq	29652653	27098004	Fig3
PTU WT, rep1	RNA-seq	37831554	31387210	Fig5
PTU WT, rep2	RNA-seq	38756185	31154045	Fig5
PTU WT, rep3	RNA-seq	37900136	29255348	Fig5
PTU WT, rep4	RNA-seq	44651817	38372446	Fig5
PTU+T3 WT, rep1	RNA-seq	40149137	25214652	Fig5
PTU+T3 WT, rep2	RNA-seq	39244876	25196277	Fig5
PTU+T3 WT, rep3	RNA-seq	37262941	21376369	Fig5
PTU+T3 WT, rep4	RNA-seq	16994596	26189084	Fig5
PTU NCORΔID, rep1	RNA-seq	50213249	40865235	Fig5
PTU NCORΔID, rep2	RNA-seq	47352437	38221861	Fig5
PTU NCORΔID, rep3	RNA-seq	53407394	42837436	Fig5
PTU NCORΔID, rep4	RNA-seq	50125641	38909396	Fig5
