# Supplementary material for: Multiple mechanisms regulate H3 acetylation of enhancers in response to thyroid hormone
Source: PLoS Genet. 2020 May 26;16(5):e1008770. doi: 10.1371/journal.pgen.1008770 (PMC7274477; doi:10.1371/journal.pgen.1008770)
Supplement: Attachment — Submitted filename: Reviewer_response_R1.pdf [file pgen.1008770.s017.pdf]

We thank the reviewer for the constructive feedback. We acknowledge comments on the text presentation of the data. As suggested by the reviewer, we have simplified many of the sentences by an introduction of abbreviations for hyperacetylated regions with a TRBS. We aimed to relate this to the current models of TR actions (type 1-4 actions) described recently (PMID: 28472304). We hope these changes to the text improves the overall readability of the manuscript. Also, we have performed new animal experiments using acute T3 treatment (2h and 6h) and performed additional H3K27Ac ChIP-seq and SRC1 ChIPs. Moreover, additional data analysis has been performed to address the concerns raised by the reviewer.

Specific response to the concerns raised by the reviewer:

Reviewer #1: The article of Praestholm et al is a genome wide analysis of mouse liver chromatin, which aims is to understand the mechanism of action of thyroid hormone (T3) and its nuclear receptors (TRs).

Overall this is a very interesting and very novel study, bringing a lot of information on a basic mechanism of gene regulation, which interest goes beyond the narrow field of T3 endocrinology. The data are quite convincing, well analyzed, and the authors reach interesting interpretations. Although the recurrent use of arbitrary threshold is risky (100 kb between TRBS and transcription start site for example), the conclusions seem reliable.

The main weakness of the study is the text presentation, which I found very difficult to follow. This is due in part to the complexity of the experiments, but the writing is also sometimes obscure. For example, the sentence line 295-299 is very long and I had to read it several times to understand it. Part of the problem is that there no abbreviation for entities like “chromatin regions in which histone acetylation is induced after T3 treatment”, and the authors might consider creating some.

We thank the reviewer for raising the issue with somewhat difficult to understand paragraphs. We have followed the advice and created abbreviations for hyperacetylated regions bound by TR. To corporate with most current models for T3 actions we have sub-grouped the type 1 actions of TR (TR bound directly to its response element on DNA, defined in PMID: 28472304) into type 1A and type 1B:

Type 1A TRBS: Bound by TR and HDAC3 in hypothyroid condition. These sites correspond to genomic regions likely kept in a hypoacetylated state by histone deacetylases in absence of hormone.

Type 1B TRBS: Bound by TR in hyperthyroid condition. Not bound by HDAC3 in hypothyroid condition. These regions are not controlled by histone deacetylases in absence of hormone.

We refer to hyperacetylated regions not bound by TR as no/TRBS in figures. Hyperacetylated regions bound by TR are referred to as w/TRBS in figures.

I have a number of questions, and suggestions for additional experiments:

1) The hypothyroid animals were treated for 5 days with T3. This is a very long time, which leaves time for a cascade of indirect effects to take place. I would recommend using a much shorter treatment, perhaps 3 hours only, and ideally to perform a time course experiment. This would help to separate causes from consequences. In particular:
- Fig3D: does TR come first, decompacting chromatin? Is histone methylation first giving access to chromatin? Or is a T3 responsive gene encoding some unknown decompacting factor?
- is the acetylation of regions which are not occupied by TR delayed, compared to the one with a TRBS? This would address the possibility that T3 induces the expression of genes encoding transcription factors able to induce chromatin acetylation at distant sites. This seems to be a good way to challenge the model showed Fig 6C.

The reviewer raises an important point. The chronic T3 treatment could indeed lead to secondary effects resulting in histone hyperacetylation not directly linked to TR activation. To address this, we have isolated livers from hypothyroid mice treated 2 and 6 hours with T3. We performed H3K27Ac ChIP-seq to quantify acute histone acetylation. We observe that 85% of the hyperacetylated regions with a TRBS are also acutely hyperacetylated. This indicates that most TR occupied regions are directly regulated by TR. We have included the data in a new supplementary figure 3 and discussed the data on page 7-8. Interestingly, we also observe that 60% of the hyperacetylated regions without a TRBS are acutely hyperacetylated in response to T3, suggesting direct histone acetylation of a subset of regions in accordance with the model shown in figure 6C. Importantly, as we do observe less regions without a TRBS acutely H3K27 acetylated, this suggests that some secondary regulation is involved. This is described on page 8 of the revised manuscript and discussed on page 19.

If we focus on hyperacetylated regions without TRBSs in super-enhancers, we observe that these regions are more likely to be acutely H3K27Ac if they are in a super-enhancer together with a TRBS. Moreover, if we focus on hyperacetylated regions without a TRBS interacting with regions containing a TRBS, we observe that these regions are also more likely to be hyperacetylated in response to acute T3 treatment. This data is included in figure 2 and discussed on page 10-11.

The question if TR comes first to a given site or if TR induces a factor that assists TR loading to chromatin is indeed interesting (especially relevant for the Type 1B TRBSs). We do not observe any obvious kinetic H3K27Ac difference between type 1A and type 1B TRBSs, suggesting that TR is directly facilitating increased enhancer activity at type 1B TRBS. We have included the data in supplementary figure 6 and discussed the data on page 12 and 18 of the revised manuscript.

2) The authors mention the classical SRC1 histone acetyl transferase coactivator, but did not perform a ChipSeq analysis for this obvious candidate. Is this for technical reason? This gap should at least be acknowledge as a limitation of the study.

We performed ChIP-seq against SRC1, however the ChIP-seq signal did not reach sufficient signal over background (Also a problem for the p300 ChIP-seqs). We have included the qPCR data on selected sites in figure 3E. We have added a comment to the lack of ChIP-seq data on page 12 of the revised manuscript.

3) The authors use two sets of ChipSeq for TRs, coming from different publications. The two datasets contain very different number of TRBS (around 1500 for Grontvedt et al and 20 000-50 000 for Ramaddoss et al , the later using a viral vector to overexpress a tagged receptor). This must affect the analysis, and the authors should explain to what extend it does.

We acknowledge the difference in the two TR ChIP-seq data sets used. Importantly, we based our score of the histone hyperacetylated regions with a TRBS solely on the endogenous TR ChIP-seq data. We have added a note in the manuscript on page 13 that states that the Ramadoss experiments are performed using TR overexpression and this may overestimate TR occupancy.

4) ChipSeq evaluate chromatin association, not DNA binding, therefore a TRBS can reflect tethering of TR by multiprotein complexes. Is this visible in super-enhancers or TADs as the models suggest? One would expect that TRBS appear simultaneously (only one with a consensus TRE) after T3 treatment at several locations. I also believe that if tethering happens, one would expect a better contrast on figure 2C, if only TRBS which contain a DR4 element had been considered.

The reviewer raises an important point. We are not able to discriminate between direct binding to DNA and association with chromatin through interaction with other transcription factors. As indicated by the reviewer, one indirect approach would be to extract TRBSs containing a consensus TRE and assume that this will represent direct binding of TR to DNA. Using this approach, it would indeed be interesting to address if contacts between hyperacetylated regions are associated with TR binding to a consensus TRE at one end and indirect TR interaction with chromatin at the other end. This could potentially be addressed with HiC data. Unfortunately, the HiC we use in this study does not capture enough interactions that allows us to study this. Future studies using for example enhancer capture HiC, where more specific enhancer-enhancer interactions are detected would be an experimental approach to address the question.

Importantly, it has previously been shown that T3 mediated histone hyperacetylation is completely disrupted in the liver of mice expressing TR unable to bind DNA (PMID: 29229863). This suggests that tethering is likely not important for hormone induced hyperacetylation. We have added a discussion of this at page 19 of the revised manuscript.

We have tried to filter the TRBS for consensus TREs (i.e. DR4 elements at different thresholds) and rescore hyperacetylated regions with a TRBS. Rerunning the overlap with super-enhancers revealed very little difference in the frequency of super-enhancers with hyperacetylated regions containing a TRBS with consensus TREs (see reviewer figure 1). This likely reflects co-occurrence of hyperacetylated regions with and without a TRBS in a given super-enhancer. After rerunning these analyses, we decided to change how we presented the overlap of hyperacetylated regions in super-enhancers (new figure 2C and 2D). We believe that this representation is more intuitive compared to the previous plot. Also, this allowed us to quantify acute H3K27Ac at regions without a TRBS within super-enhancers (new figure 2E). This new way of plotting the data is also more comparable to the new way we did the analysis of TRBS in TADs shown in figure 2H.

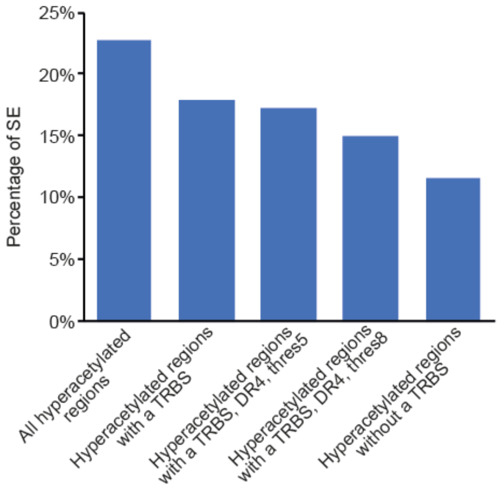

Review Figure 1. Frequency of superenhancers (SE) occupied by hyperacetylated regions with TRBS or no TRBS. TRBS are filtered for presence of DR4 scored at two different thresholds. Log odds motif score of a least 5 (DR4, thres5) and 8 (DR4, thres8).

5) Figure S3 motif search: I suppose that if the DR4 is actually enriched, one should also find enrichment for the half-site (6mer), and thus, accidentally, for other half-sites organization. Therefore it seems that there is no reason to believe that other nuclear receptors are actually present. This should be clarified.

We agree with the reviewer that the half site should be present in the motif analysis. After browsing through identified motifs with high similarity to the DR4, we do indeed find enrichment of the DR4 half-site. We have now included the DR half site in supplementary figure 4F. Like all other non DR4 motifs, the DR half motif is not enriched more or less comparing the hyperacetylated regions with and without TRBS. This suggests that other nuclear receptors may be present, but the presence seems not to explain T3 mediated hyperacetylation in absence of a TRBS.

6) I suspect that there is a continuum, (ie no clear threshold) between poised and dormant enhancers (especially because the number of detected TRBS is highly variable from one experiment to the other and dependent on several statistical thresholds). Perhaps the use of other available TR Chipseq datasets (heart, cell line) could help to ask whether TR requires cell-specific factors to access the DR4 elements, and whether this explains why different cell types activate different genes in response to T3.

We agree with the reviewer that there is a continuum between poised (type 1A) and dormant enhancers (type 1B). Likely as a consequence of the variable experimental and computation approaches used to identify TRBSs. We aimed to analyze the extremes in this continuum to emphasize the different modes of action. We certainly believe that TR require different transcription factors to access chromatin depending on the cell type. We believe that this would be an interesting analysis when more and more TR ChIP-seq datasets become available. However, we think this would be out of the scope of this particular manuscript.

7) After reading, one has no response to simple questions: how much of the transcriptional response to T3 is explained by the canonical model, and the alternative models presented by the authors? And how much remains unexplained? Previous studies reported different modes of T3 response for different gene clusters (Yen PMID:12776178; PMID:29229863 Hönes

PMID:29229863). Can this be related to the present models? Fig5A indicates that only half of the T3 induced genes are associated with hyperacetylation, while Fig5b 30% of these have no proximal TRBS. This leaves only 35% of the T3 induction explained by the presence of a proximal TRBS. In general, the relationship between chromatin status and gene expression should be better discussed.

Linking gene expression to regulated enhancers remains a challenge. We acknowledge that the manuscript does not comprehensively discuss how much of the T3 induced gene expression that can be linked to the different types of histone hyperacetylation. To accurately link histone hyperacetylation with gene expression we need deep sequenced HiC promoter capture data for the hypo- and hyperthyroid animals. Unfortunately, we do not have this kind of data and the resources to generate the data. Therefore, we use the 100kb proximation approach to link hyperacetylated regions to gene expression. This has obvious limitations but provides some correlative information on histone hyperacetylation and gene expression:

a) Histone hyperacetylation by T3 is associated with T3-induced gene expression and not with T3 repressed gene expression.

b) Less than half of the T3-induced genes are associated with a nearby hyperacetylated regions, suggesting that many T3-induced genes may be regulated by mechanisms not necessarily involving histone hyperacetylation of enhancers. Alternative, genes may be controlled by enhancers more than 100kb from the TSS. We need high resolution HiC promoter capture data to answer this question. We have included a discussion of this at page 20 of the manuscript.

c) We observe that induced genes are associated with hyperacetylated regions with and without a TRBS, suggesting a gene regulatory functions of both types of hyperacetylated regions.

d) We tested how many of the T3 induced genes, associated with a hyperacetylated regions, are controlled by NCOR recruited to TR (Figure 5B) – representing the canonical model. This showed that only a minority of the T3 induced genes are controlled by NCOR recruited to TR. Also indicated in the Yen PMID:12776178 study. This is also discussed on page 20 in the revised manuscript.

8) I suspect that if a 100 kb threshold distance is used, as the authors did, most if not all genes might have a proximal TRBS. In that case it is difficult to understand why only 1015 genes are upregulated by T3. Should the TRBS and the gene be in the same TAD? It would be interesting to consider numbers in the other direction: among all the DR4 consensus found in the genome, which fraction is occupied by TR before and after T3 treatment? Among those, which ones are occupied by NcoR in hypothyroid mice, and Med1 is T3 treated mice? Is the T3 response of a gene always accompanied by hyperacetylation of the nearby chromatin? how many genes are close to a gene which is T3 responsive?

Out of 31.370 annotated genes in the mouse genome 2434 genes are associated with a hyperacetylated region containing a TRBS (within 100kb of the TSS), meaning that less than 8% of the genes in the genome is associated with a proximal TRBS. Thus, close to half of the TRBS

associated genes are induced by T3. The other half remaining unresponsive to T3 may be epigenetically silenced or lack 3D connection to the hyperacetylated region.

We acknowledge that an analysis centered on DR4 motifs would be interesting. Previous studies show that the DR4 element is enriched in TRBS associated with ligand dependent TR recruitment and in TRBSs where TR is bound in absence of hormone (PMID: 25916672 and PMID: 24288132). In fact, TRBS associated with de novo chromatin remodeling (i.e. typical type 1B TRBS) are particular enriched for the DR4 motif (PMID: 25916672). This suggests that the DR4 motif is not a factor that explains the difference between TRBSs. Also, we find that the acute treatment with T3 leads to histone acetylation at the vast majority of type 1B TRBSs arguing that these TRBSs are direct TR binding sites.

When we correlate histone hyperacetylation with gene expression we observe that about half of the T3 induced genes have hyperacetylated regions within 100kb of the TSS. This is not observed for T3 repressed genes, suggesting a good correlation between induced gene expression and histone hyperacetylation. Yet, the 100kb approximation approach does not support the idea that all T3 induced genes are explained by nearby histone hyperacetylation. This could be explained by the obvious limitation of the approach used and the possibility that genes are controlled by regions positioned more than 100kb from the TSS. Alternatively, genes may be controlled independent of histone hyperacetylation.

I believe that it would be in the authors interest to perform at least some of the suggested experiments. In conclusion, although I found the reading difficult, I am convinced that the authors made a substantial progress in the understanding of gene regulation by T3, in a relevant physiological system, which is a very significant achievement.

Other points:

Line 179 or 767: How is exactly made the random selection?
Random selection was performed in R using Sample() function. We have added an explanation in the methods section.

Line 194: is it possible to make this statement statistically significant?
We have changed the analysis and compared to random selected regions. New figure 2H.

Fig 2F: subTAD is not defined and the interpretation obscure. This might require further explanation.
SubTADs have been defined on page 10 of the revised manuscript.

FigS3F1: I believe that this is DR4 not DR1
It certainly is, we apologize for this mistake. This have been corrected in the revised manuscript.

Fig 2H: I do not really followed the explanation in the legend and the text. Perhaps this could be clarified.

HiC quantify interactions between specific regions, however quantification can be confounded by distance. I.e. interaction over longer distances are more likely to be scored significant compared to shorter distance interactions. To control for any potential background interaction in the HiC dataset we quantified interactions in the opposite direction in the genome. If this shows equal intensity this would indicate that interactions are merely background. We have modified the text describing the figure and the figure legend. We hope this helps to understand the analysis.

Fig 3C: Unless I missed it, the legend or the method section do not explain how this heatmap was generated.
We apologize for omitting this information. We have included the details on heatmap analysis to the methods section.

Fig 3E: the genomic coordinates of the amplified fragments should be given somewhere.
The genomic coordinates are indicated in table S2. We have added this information in the figure legend.

Fig 4H: the genomic coordinates of these regions should be indicated in the legend (and the possible presence of genes).
We apologize for omitting this information. The genomic coordinates have been added to the figure legend.
